# National Hospital for the Paralysed and Epileptic

**Published:** 1904-11-19

**Authors:** 


### NATIONAL HOSPITAL FOR THE PARALYSED AND EPILEPTIC.

A special general meeting of Governors and members of this hospital was held on Tuesday, 15th inst., Mr. J. Danvers Power, Chairman of the Board of Management, presiding.

Mr. Danvers Power pointed out that the year 1899 was the last year in which the hospital was able to pay all its expenses out of its yearly income. The deficit in the year 1900 was £3,201, and in 1901 £3,854, which only left £687 in hand on January 1st, 1902. The deficit in 1902 was £5,569, in 1903 £7,022, and in 1904 it was £4,781. Consequently, the sum of £17,374 had to be accounted for. Of this sum £9,576 had been spent on what might be called capital expenditure, as it had been absolutely necessary to make certain improvements in the sanitary and heating arrangements of the hospital, as well as in the fire escapes, electric lighting, the new operating theatre, and other matters which had been considered essential. Consequently there only remained £6,798 as the deficit on the ordinary accounts of the last three years, or something like £2,115 per annum. But this deficit would have been much greater had it not been for a very exceptional legacy, and such legacies could not be counted upon in the future. Indeed, the regular income of the hospital was only £13,000, while the expenses were £19,000, thus leaving a deficit in the future of £6,000 a year to be dealt with. It was proposed to borrow £10,000 to repay the bankers' loan, which, with the sale of stock and one or two other small items, would only leave a balance of £2,543 to be provided, and that they could borrow on the security of stock which was free from any trust.

A resolution was proposed by Sir John Paget, seconded by Dr. T. Buzzard, and carried unanimously, to the effect that a loan should be raised, with the consent of the Charity Commissioners, on the security of the Hospital's buildings and land. A discussion ensued as to what part of the Hospital's work would have to be discontinued in order to deal with future deficits, and Dr. Buzzard, in the course of a few remarks, said that it would appear to him, on the whole, desirable to close the Convalescent Home at Finchley, rather than to curtail the activities of the Hospital in any other direction.

